# A Wireless Interface for Replacing the Cables in Bridge-Sensor Applications

**DOI:** 10.3390/s120810014

**Published:** 2012-07-25

**Authors:** Marko Pavlin, Franc Novak

**Affiliations:** 1 In.Medica d.o.o., Levicnikova 34, 8310 Sentjernej, Slovenia; 2 Jozef Stefan Institute, Jamova 39, 1000 Ljubljana, Slovenia; E-Mail: franc.novak@ijs.si

**Keywords:** bridge-sensor measurements, wireless interface, reciprocal topology, sensor data integrity and reduction, comparative test methods, signal stream delay testing

## Abstract

This paper presents a solution in which a wireless interface is employed to replace the cables in bridge-sensor measurement applications. The most noticeable feature of the presented approach is the fact that the wireless interface simply replaces the cables without any additional hardware modification to the existing system. In this approach, the concept of reciprocal topology is employed, where the transmitter side acquires signals with its own transfer function and the receiver side reconstructs them with the transfer function reciprocal to the transmitter transfer function. In this paper the principle of data acquisition and reconstruction is described together with the implementation details of the signal transfer from the sensor to the signal-monitoring equipment. The wireless data communication was investigated and proprietary data-reduction methods were developed. The proposed methods and algorithms were implemented using two different wireless technologies. The performance was evaluated with a dedicated data-acquisition system and finally, the test results were analyzed. The two different sets of results indicated the high level of amplitude and the temporal accuracy of the wirelessly transferred sensor signals.

## 1. Introduction

Cable connections can lead to difficulties in many sensor applications [1–3]. The key advantage of using wireless technologies in industrial, medical, environmental and other sensor applications is that they can significantly improve the flexibility and lower the costs associated with installing, maintaining, and upgrading wired systems [4,5]. The commercial promise of such solutions has been proved by the vast number of installed applications [6] based on new standards, like IEEE802.15.4 [7], and industrial collaborations, like the Zigbee Alliance [8]. All these new installations and wireless sensor applications, which are often very application specific, require a new infrastructure or at least some degree of adaptation to implement the wireless sensor technology in industrial or other environments. Any adaptations or new installations may result in significant investments. Some specific environments are very sensitive with regard to the investments, and the costs of going wireless are not always justified [9,10]. A good example is hospitals, where sensors are used in intensive care. All those sensors, placed on or near the patient, are traditionally connected with wires to the monitoring equipment [11]. However, monitors, even modern ones, have no standard wireless interfaces to connect to the sensors and the introduction of wireless sensors would require the replacement of the monitors [12,13].

In this paper we propose a solution in which a wireless sensor interface is employed to replace the cable connection of an existing measurement system. The salient feature of the proposed approach is that no adaptation or change to the sensor or to the measuring-equipment site is required. We developed reciprocal transmitter and receiver architecture which enables simultaneous multiple monitoring of a sensor signal. In a running system, only the data at the receiver are available, and the delay cannot be estimated. To solve this problem, a dynamic time-adaptation algorithm was developed. In the following we outline the advantages of the proposed topology and the requirements for its real life applications. In the second part of the paper we describe implemented prototypes and their operation in practice. The developed dynamic time-adaptation algorithm is demonstrated in the description of the signal time delay test evaluation.

While the presented solution can be applied to different passive bridge-sensor implementations and does not depend on a target application, we illustrate the advantage of the proposed approach on a case study of a blood-pressure monitor as a typical medical application. Test results were collected during clinical evaluation, which proved the commercial system based on presented concepts met the requirements of IEC-60601-1-2:34 {ed. 2.0} and base of the usability's requirements of the IEC 60601-1-6_2010 ed.3 and IEC 62366_2007 ed.1. The clinical trial was conducted under identifier NCT01373996.

## 2. Bridge-Sensor Connections

Bridge resistors are primarily sensitive to the primary measured parameter (e.g., the pressure). The output signal, however, is also influenced by other parameters (e.g., the temperature), generating an error in the differential output voltage, which can be minimized by a compensation circuit or an algorithm. There are different implementations of bridge sensors. Amplifiers or even a complete microcontroller with temperature compensation and calibration can be integrated within a sensor. In this paper we focus on a Wheatstone-bridge configuration, which is common in many sensors operating on a resistance-change principle. They are (but not limited to) the following: piezoresistive pressure sensors, strain-gauge sensors, temperature bridge sensors, and many more.

**Figure 1.**
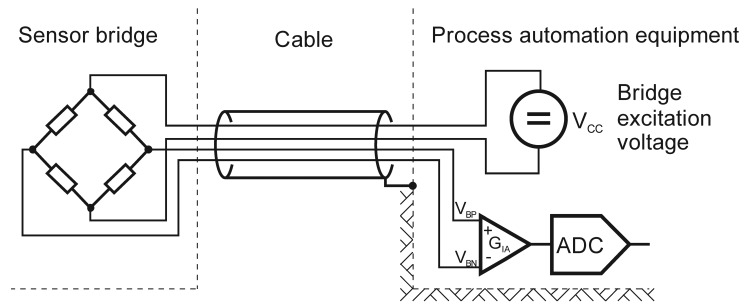
Passive sensor bridge connected to the process equipment.

A conventional bridge sensor is connected to the monitoring or measuring equipment with a minimum of a four-lead cable, as shown in Figure 1. The cables that are placed between the sensor and the process-automation equipment are sometimes difficult to install. Another limitation is the case when one process requires more than one measuring channel per sensing parameter. Such a case would require connecting one sensor to two instruments, which is not feasible with passive bridge sensors. The supply lines are either in short circuit, or the sensor bridge is not supplied by the correct instrument, which results in an incorrect readout. The above problems can be solved by replacing the cable with a wireless communication, as described in the following sections.

In most cases the equipment is a part of an existing infrastructure that is already installed, or there is a need to upgrade the system with some standard, additional instrumentation. Both cases have one common feature: the only accessible points are the external connections of the bridge sensors and the measurement instruments. Usually, there is no possibility to adapt the internal structure of the instruments to directly implement the use of any kind of wireless technology. The proposed wireless resistive-bridge sensor interface is suitable for a cable replacement in the existing systems and requires no modification of the measurement infrastructure.

## 3. Wireless Cable-Replacement Interface for Bridge Sensors

**Figure 2.**
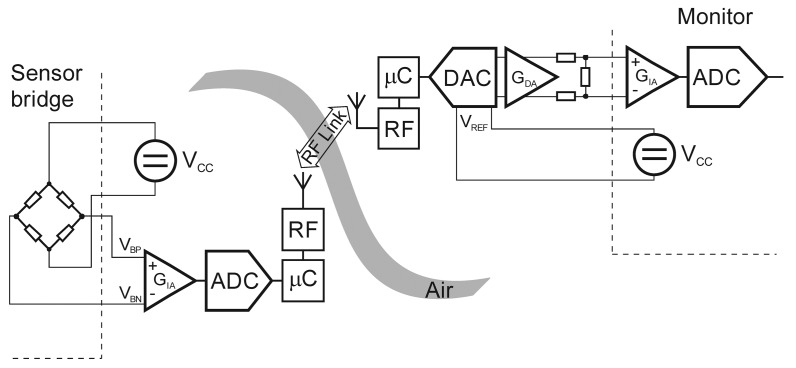
Wireless resistive-bridge sensor interface-block diagram.

The wireless resistive-bridge sensor interface integrated in an industrial or medical sensor system consists of transmitter and receiver units connected with a secure RF link within the unlicensed Industrial, Scientific and Medical (ISM) frequency band at 2.4 GHz (Figure 2).

A bridge-sensor element has a low differential voltage output. One typical example is a ceramic pressure sensor [14]. Its sensitivity is in the range of mV/V at a full-scale input (ratio 1:1,000) [15,16]. Such low-level voltages introduce two issues that are specific to a wireless system.

First, at the transmitter side, the mV range from the sensor bridge is too low for the Analog-to-Digital Converter (ADC), which requires higher input-voltage levels, which means an amplifier with a gain G_IA_ needs to be placed at the ADC converter input. The digital output stream from the transmitter is thus proportional to the amplified voltage, not to the mV output from the sensor bridge. In the subsequent discussion, however, let us denote the ratio between the transmitter output and the low-level sensor signal as the transmitter gain G_TX_.

The second issue is related to the receiver side. The Digital-to-Analog Converter (DAC) has an output voltage range from −V_REF_ to +V_REF_. The reference voltage V_REF_ has its lower limit, usually in the 1 V range. The received digital stream is fed to the DAC in the receiver unit. The transmitter ADC and the receiver DAC have the same resolution and operating range. Their digital ports are linked via the wireless interface. Ideally, the DAC will generate an output voltage, which is equal to the ADC input voltage (with equal reference voltages). To reconstruct the low voltage level at the receiver output, which is equal to the sensor-bridge output voltage, the receiver must have an attenuation that is reciprocal to the transmitter gain. If the receiver gain is represented by G_RX_, then the following condition must be met: G_TX_ = (G_RX_)^−1^.

As shown in Figure 2, the wireless interface requires an additional signal-acquisition module with a sensor-bridge supply, two RF link modules and a signal-reconstruction module. The excitation voltage for the resistive sensor bridge is supplied by the transmitter unit. The sensor differential output voltage is amplified by an instrumentation amplifier (G_IA_), digitized by an ADC and processed by a microcontroller (μC). The wireless link is based on a standard wireless technology (Bluetooth, IEEE 802.15.4) with standard or proprietary protocols. The processed readouts are transmitted to the receiver side, which is connected to the measuring instruments or process automation equipment. The receiver RF link passes the received readouts to the microcontroller. The receiver unit mimics the sensor bridge with a multiplying DAC. The output voltage from the multiplying DAC is proportional to the digital word multiplied by the reference voltage. It operates in all four quadrants with negative or positive DC or AC reference voltages.

The described wireless interface provides an efficient solution to the problems mentioned above. It does not require any modification within the internals of the process equipment. Furthermore, one sensor can be connected to several measurement channels because there is only one sensor excitation. The operation of the proposed wireless sensor interface is described in the following sections.

### 3.1. Transmitter Data Acquisition

**Figure 3.**
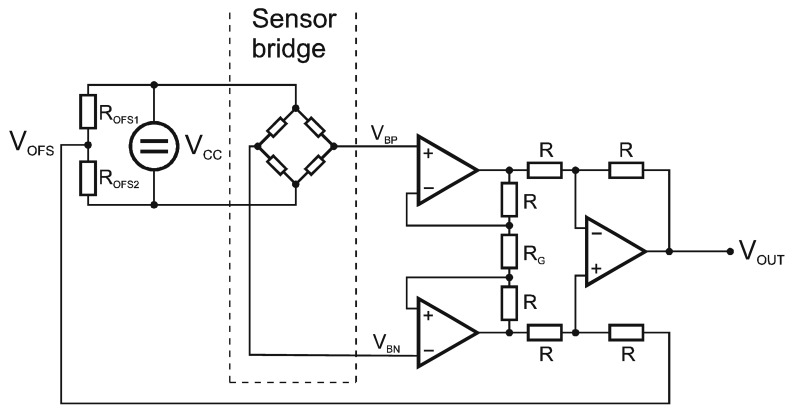
Analog input stage at the transmitter side.

The transmitter side of the wireless interface is connected to the resistive sensor bridge. The interface also provides an excitation voltage for the pressure transducers (Figure 3). Let the sensor-transfer characteristics be represented by the transfer function *f(p)*. The sensor bridge has a differential output voltage in the mV range, ratiometric to the supply voltage V_CC_ (Equation (1)):

(1) $$V_{P}=V_{BP1}-V_{BN1}=V_{CC}f(p)$$

The output voltage *V_OUT_* from the instrumentation amplifier is the sum of the amplified differential input voltage *V_P_*, multiplied by the instrumentation amplifier gain *G_IA_*, and the offset voltage *V_OFS_*:

(2) $$V_{\text{OUT}}=G_{IA}V_{P}+V_{\text{OFS}}$$

The instrumentation amplifier gain *G_IA_* is determined and fixed by the resistor *R_G_*. The offset voltage is proportional to the supply voltage and defined by the resistor divider *R_OFS1_* and *R_OFS2_* (Figure 3).

(3) $$V_{\text{OFS}1}=V_{CC}\frac{R_{\text{OFS}2}}{R_{\text{OFS}1}+R_{\text{OFS}2}}$$

The amplifier output voltage is connected to the ADC. The reference voltage *V_REF_* is derived from the supply voltage by the resistor divider *R_OFS1_* and *R_OFS2_* and is equal to the offset voltage *V_OFS_*:

(4) $$V_{\text{REF}}=V_{\text{OFS}}=V_{CC}\frac{R_{\text{OFS}2}}{R_{\text{OFS}1}+R_{\text{OFS}2}}$$

**Figure 4.**
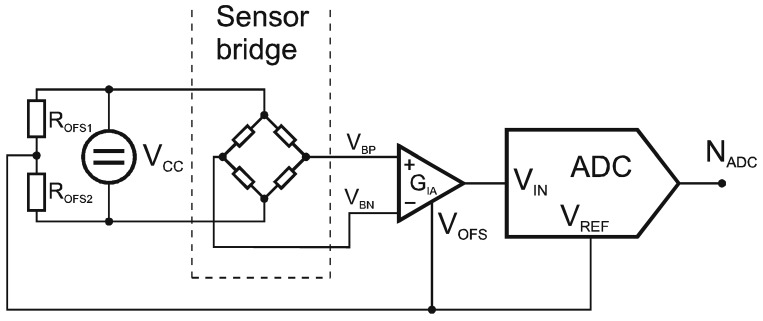
Analog-to-digital converter connection.

The 16-bit digital output value from the ADC is proportional to the ratio of the ADC input voltage and the reference voltage (Figure 4):

(5) $$N_{\text{ADC}}=2^{16}\frac{V_{\text{INADC}}}{V_{\text{REF}}}$$

Replacing *V_INADC_* = *V_OUT_* we obtain:

(6) $$\begin{array}{l} N_{\text{ADC}}=2^{16}\frac{G_{IA}V_{P}+V_{\text{OFS}}}{V_{\text{REF}}}=\\ N_{\text{ADC}}=2^{16}\frac{G_{IA}f(p)+\alpha }{\alpha } \end{array}$$

where:

(7) $$\alpha =\frac{R_{\text{OFS}2}}{R_{\text{OFS}1}+R_{\text{OFS}2}}$$

Equation (6) has no reference voltage, which means the ADC reference voltage could be an arbitrary value as long as it is within the ADC specification required by the ADC. This is true only when the sensor-bridge excitation and the ADC reference voltage are proportional or equal (Equation (4)). Finally, the digital readouts *N_ADC_* are buffered and transmitted via a wireless link (Figure 4).

### 3.2. Receiver Data Reconstruction

**Figure 5.**
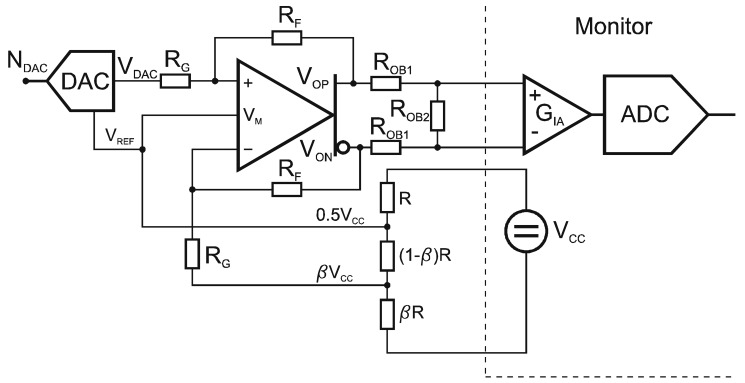
Output stage of the sensor signal reconstruction unit: the main component is the fully differential amplifier.

Digital readouts are transmitted to the receiver. The raw digital data *N_DAC_* feeds the digital-to-analog converter with the same sampling rate as the ADC converter in the transmitter. The applied external reference input voltage *V_ref_* determines the full-scale output voltage of the DAC converter:

(8) $$V_{\text{DAC}}=\frac{N_{\text{DAC}}}{2^{M}}V_{\text{REF}}$$

(9) $$V_{\text{REF}}=V_{CC}\frac{R}{\beta R+(1-\beta )R}=\frac{1}{2}V_{CC}$$

where 2^M^ refers to the DAC resolution. The reference voltage is set to one half of the bridge supply voltage (V_CC_/2). The DAC output is connected to a fully differential amplifier. It has a differential input and a differential output (Figure 5).

The differential output voltage is generated by the differential driver with a rail-to-rail output. Its internal common-mode feedback architecture allows its output common-mode voltage to be controlled by the voltage applied to *V_M_*. To implement the fully differential configuration the feedback resistors and attenuator resistors must match perfectly. One option is to select the resistors and sort them into several bins, which creates many problems when it comes to mass production. Another option is to use high-precision resistors, which raises the cost of the implementation. An optimal trade-off between the costs, the mass-production requirements and the performance could be implemented with a thick-film hybrid-circuit technology providing the ultimate match of the feedback network consisting of four resistors that determine the amplifier's closed-loop gain. The amplifier's differential output voltage (Figure 5) is defined as:

(10) $$V_{\text{ODIF}}=V_{OP}-V_{ON}$$

The common-mode output voltage is the average of the two voltages and is defined as:

(11) $$V_{\text{OCM}}=\frac{V_{OP}+V_{ON}}{2}$$

The voltage gain of the single-ended-to-differential output topology can be derived from the signal definitions shown in Figure 3. The setting *V_OFS2_* = 0 can be written as:

(12) $$\frac{V_{\text{DAC}}-V_{AP}}{R_{G}}=\frac{V_{AP}-V_{ON}}{R_{F}},V_{AN}=V_{AP}=V_{OP}\left(\frac{R_{G}}{R_{F}+R_{G}}\right)$$

Solving the above Equation (10) gives the gain relationship for *V_ODIF_/V_DAC_*:

(13) $$V_{OP}-V_{ON}=V_{\text{ODIF}}=V_{\text{DAC}}\left(\frac{R_{F}}{R_{G}}\right)$$

An inverting configuration with the same gain magnitude can be implemented by simply applying the input signal to *V_OFS2_* and setting *V_DAC_* = 0. For a balanced differential input, the gain from *V_DAC_* − *V_OFS2_* to *V_ODIF_* is also equal to *R_F_/R_G_*.

The advantages of the proposed topology can be summarised as follows:

- there is no need for accurate (and costly) voltage references in the transmitter or receiver,
- an additional receiver or transmitter calibration is not needed with a mindful circuit design,
- the signal reconstruction operates equally as a Wheatstone bridge in all four quadrants.

A disadvantage of the presented solution is that it requires a highly matched resistor network. There are other types of amplifiers, which can be deployed in this circuit and are commonly used. One is differential amplifier implemented with singe operational amplifier. True instrumentation amplifiers with three or two operational amplifiers may be implemented. They all amplify differential signals with good rejection of common mode input voltages. The latter two are popular for discrete implementation having lower requirements on resistors. Since our amplifier circuit is implemented in a thick film hybrid circuit technology, the resistor matching is not an issue, because they are all precisely laser trimmed.

### 3.3. Data Reduction

The performance of a battery-operated wireless device depends on the energy efficiency. One of the main consumers of the energy in the battery is the RF link. The transmitter's power efficiency could be maximised by minimizing the RF link's active time. The active time starts with the transition from a low power state to the active state. This period is followed by a data-transmission interval. Finally, the RF link needs some time to switch from the active mode back to the sleep mode. The transition intervals are significantly shorter than the active transmission. The main focus in minimizing the RF link's power consumption should be during the active period. The easiest way to reduce the power consumption of the RF transmitter is to reduce the bandwidth of the RF link by sending less data without losing performance.

During the system design, it is necessary to define the required system resolution δ_X_ and the operating range (*x_MAX_*−*x_MIN_*). Both parameters define the minimum number of bits required to store any value within the operating range:

(14) $$M_{S}>\log_{2}\frac{x_{\text{MAX}}-x_{\text{MIN}}}{\delta_{X}}$$

where *M_S_* is the smallest number of bits.

Another possible solution to reduce the amount of data being transmitted is data compression. However, the lossless compression of a continuous data stream is a challenging task for a resource-limited embedded microcontroller. The compression method should conform to the restrictions imposed on the processing power and the memory resources. The best method to reduce data rate is to use a custom data format with a resolution equal to *M_S_*. The data reduction is then implemented as simple bit-manipulation, where all the processing remains within the microcontroller register file. In our implementation we used half-precision 16-bit floating-point numbers using 1 sign bit, a 5-bit excess-15 exponent and 10 mantissa bits, in accordance with IEEE Std 754-2008 [17].

### 3.4. Data Integrity

**Figure 6.**
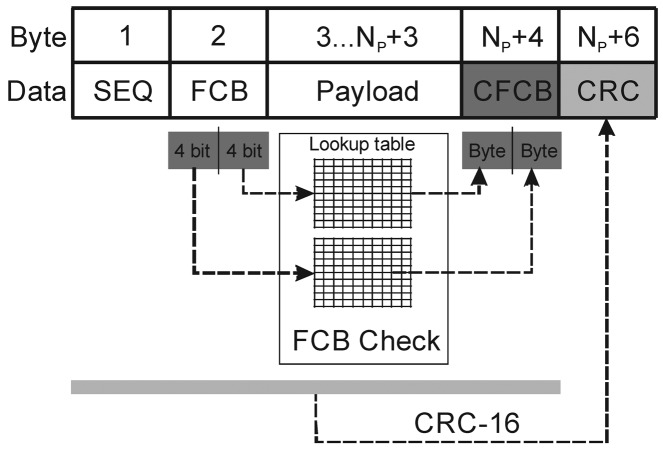
Data-integrity protection envelope.

Replacing a cable with a wireless interface presents new challenges for the field of data integrity. It is not uncommon to be faced with issues relating to the integrity of readouts transmitted via a wireless interface. This wireless interface is usually small in size and has limited computing capabilities. With some safety-critical applications, like healthcare, data integrity ranks high in the core requirements. Wrong decisions caused by invalid data may harm someone's health or even result in the death of a patient. One such example is wireless blood-pressure monitoring. When used, e.g., in a critical-care unit, many decisions are based on instruments' readouts. If the blood pressure displayed by the patient's monitor is wrong, the patient could receive the wrong treatment, resulting in a critical state. In such cases, it is better not to transfer any data at all than present data that might be invalid. With this in mind, three levels of error detection were implemented: sequence-number checking, control-byte error checking and the checksum calculation. The whole data frame structure is shown in Figure 6.

At the upper level, a sequential freshness security service is employed. Sequential freshness maintains an ordered sequence of received frames. When a frame is received, the freshness value is compared with the last known freshness value. If the freshness value is newer than the last known value, the check has passed, and the freshness value is updated to the new value. If the freshness value is not newer than the last known freshness value, the check has failed. This service provides evidence that the received data are newer than the last data received by that device.

The frame control byte (FCB) represents the essential part of an application frame. The bit pattern within the FCB defines the type of payload or serves as a special command, like data-acknowledge, data-acquisition start and stop, *etc.* The FCB is divided in two 4-bit symbols. The FCB error detection is provided with 4-bit to 8-bit coding. There are only 16 valid byte codes for each 4 bit half of the FCB. Each symbol is coded with an 8-bit code. Finally, the check sum calculation is operated over the complete frame and added at the end of the frame. Each data frame has control structures with a data payload (Figure 6). Some special frames such as reception acknowledge or command strobes have no data payload.

## 4. System Evaluation

### 4.1. Implemented Prototypes

**Figure 7.**
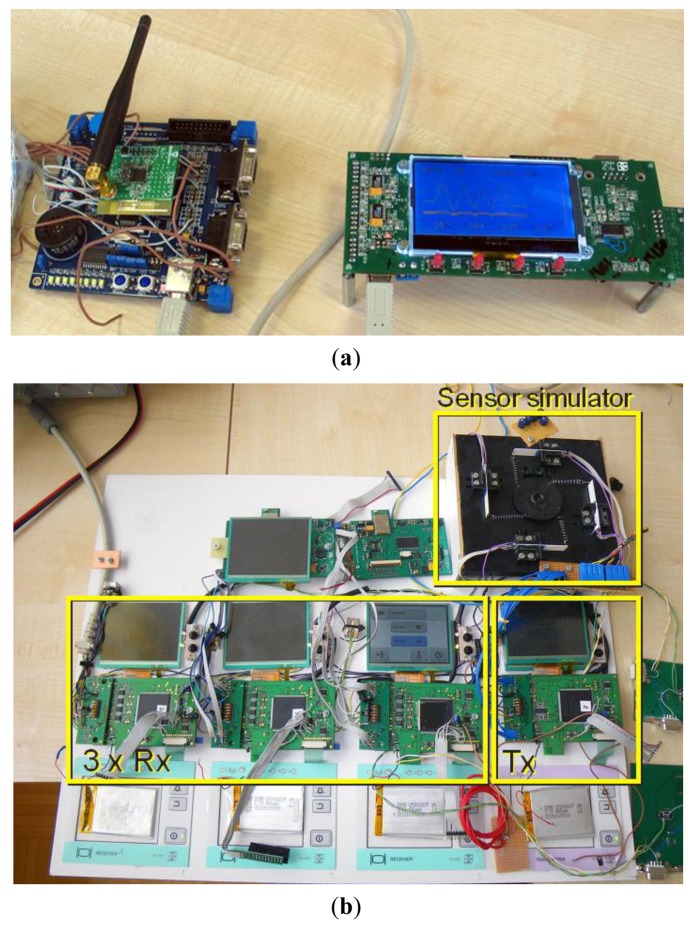
(**a**) Transmitter and receiver prototype implemented with CC2400 and (**b**) Transmitter and three receivers implemented with WT12 Bluetooth module.

The wireless-sensor interface shown in Figure 2 was implemented with two different wireless technologies. The first set was implemented on IEEE 802.15.4 transceivers based on the CC2400 from TI/Chipcon (Figure 7(a)). The second pair was implemented with standard Bluetooth WT12 modules from Bluegiga (Figure 7(b)).

The main reason for the implementation with two different wireless technologies was their evaluation for industrial and medical applications. They are both some of the most commonly used in a variety of applications. IEEE 802.15.4 is preferred in industrial applications, like ZigBee and similar protocols. Medical applications are more Bluetooth oriented.

The following table presents the main characteristics of the two wireless technologies that are relevant for the selection of a target application. The data gathered in Table 1 could be used as a guideline when selecting the optimum wireless technology. The final decision, however, also depends on other factors, like availability, legal and safety compliance with medical or industrial applications, *etc.*

### 4.2. Evaluation Procedure

**Figure 8.**
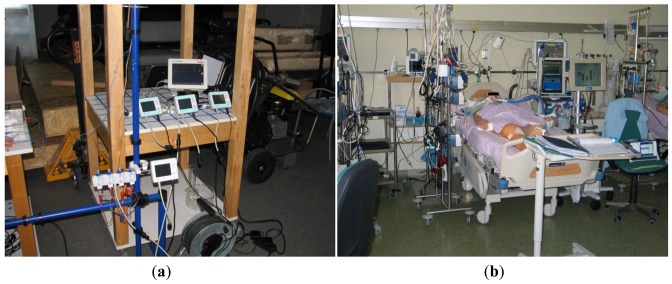
(**a**) Clinical evaluation setup under laboratory conditions and (**b**) during clinical trial in the hospital intensive care unit.

**Figure 9.**
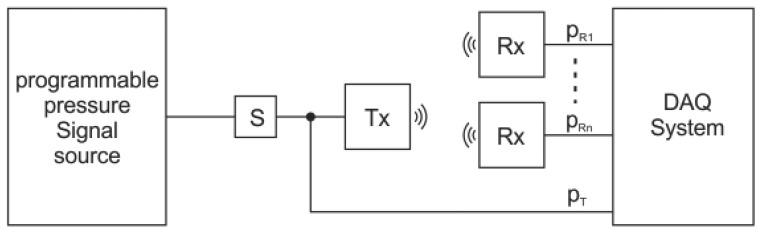
Evaluation data-acquisition system.

In order to evaluate the performance of the developed wireless interface for replacing cables in bridge-sensor applications, a dedicated data-acquisition system was constructed. The clinical evaluation system (Figure 8) schematic is shown in Figure 9. The pressure was generated using a programmable pressure source for laboratory testing, while real live signals from different patients were taken during clinical trial. The calibrated pressure sensor S was used for the reference electrical signal. The output voltage from sensor S was connected to the transmitter unit and served as a reference signal *p_T_*. The same signal was directly connected to the data-acquisition system. The transmitter output signal was wirelessly transmitted to several receiver units simultaneously. All the receiver signals *p_R1_, p_R2_ p_RN_* were measured with dedicated channels in the DAQ.

The purpose of the study was to determine whether the wireless transmission of pressure signals from the sensor to the data-acquisition system is feasible, safe and as accurate as a conventional cable connection. The analysis was made by comparing pressure-signal pairs *p_T_* and *p_Ri_* (*i* = *1, …,N*). Each signal was sampled and stored in files over a long period of time. The sampling frequency was twice the sampling frequency of the wireless interface, which resulted in huge data files. The gathered data was, therefore, analyzed offline.

**Figure 10.**
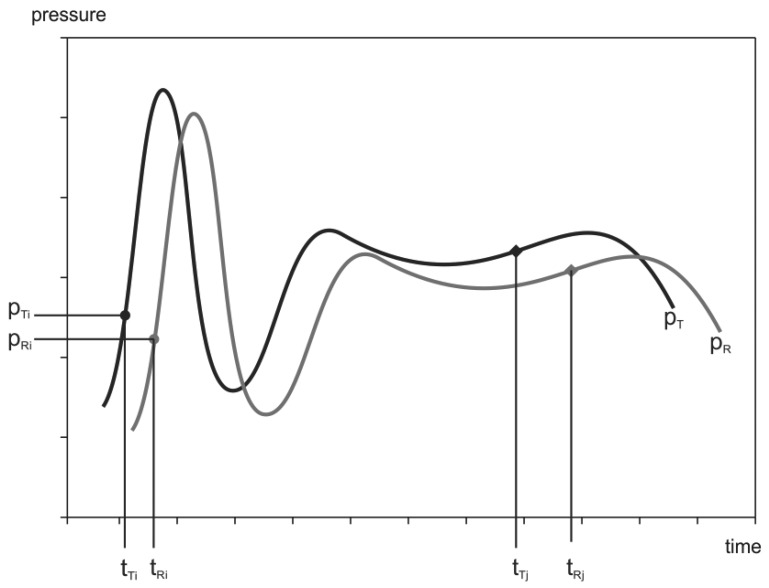
Signals captured at the lower level of the experiment.

As shown in Figure 10, the signal *p_R_* is delayed after the signal *p_T_*. The wireless-data connection is not ideal; it exhibits some error in the time and the amplitude. In addition, the delay time and the amplitude error between the signals may change over time [18]. The main reason for the time-delay variability is the difference in the internal clocks between the transmitter and the receiver unit (Figure 10). The amplitude error is caused by component tolerances in the transmitter acquisition and receiver reconstruction circuits.

### 4.3. Testing Method

**Figure 11.**
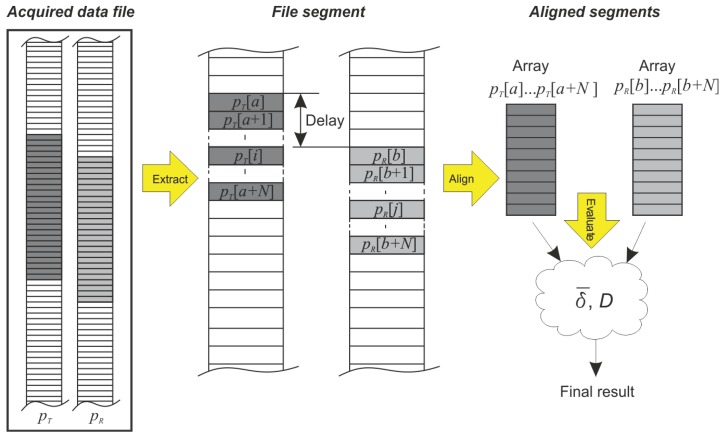
Data-extraction and evaluation algorithm.

The wireless interface was measured with the DAQ system over a one-day period. As mentioned above, the measurement resulted in an enormous amount of data that was stored in huge files. It was not possible to perform the evaluation on a sample-by-sample basis [19]. To handle such long data records, it is necessary to split the long data record into segments of a manageable size. In order to evaluate the quality of the wireless data transmission a dedicated, multiple-step data-extraction and evaluation algorithm was developed. The principle of the algorithm is sketched in Figure 11.

### 4.4. Data-Extraction and Evaluation Algorithm

The acquired test-results file contains two sets of data: *p_T_* from the transmitter side and *p_R_* from the receiver side. Both signals were sampled simultaneously, although they are not correctly aligned due to the delay induced by the wireless system. The first step in the algorithm is the extraction of segments that are suitable for analysis. The segment size was determined in a preliminary analysis that considered two criteria. The first one was the trade-off between the number of segments and the computational speed: shorter segments require a shorter processing time. On the other hand, this would result in a larger number of segments, which is more difficult to handle. The second criterion was related to the duration time of the segmented data. The programmable pressure source generated a periodic signal. To keep the analysis consistent at least two input signal periods should be present in a single segment. Consequently, one whole period of the transmitter periodic signal *p_T_* lies between the segment array indexes *a* and *a* + *N*, as shown in Figure 11.

The corresponding samples appear in the receiver signal *p_R_* between indices *b* and *b* + *N* as an approximate and a delayed approximate of the signal *p_T_*. In order to calculate the difference between the two signals, they must be aligned in time. This is done by shifting the output signal back until the shapes fit. In order to determine the actual error induced by the wireless system, the two signals must be perfectly aligned. The index *b*, which represents the start of the transmitted signal within the received array, is not known. The difference between the indices *a* and *b* (see Figure 11) characterizes the time delay and is determined by calculating the cross-correlation between the two signals.

Let the extracted segments of the signals *p_T_* and *p_R_* be represented by the arrays:

(15) $$p_{T}\left[i\right]=\left[p_{Ta},p_{Ta+1},\ldots ,p_{Ta+N}\right]$$

and:

(16) $$p_{R}\left[j\right]=\left[p_{Rb},p_{Rb+1},\ldots ,p_{Rb+N}\right]$$

where *a* is the starting index and *N* is the segment length limiting the transmitter-segment boundaries. The corresponding segment in the receiver signal is denoted by the index *b* and the equal length *N* (see Figure 11).

The array segment within the receiver signal array is shifted back in time:

(17) $$p_{R}^{\prime }\left[j\right]=p_{R}\left[j-k\right]$$

where the array shift is denoted by the lag difference *k*. The maximum *k* is related to the buffering mechanism of the wireless system and is proportional to the buffer width. The signal delay is determined by calculating the normalized cross-correlation between the segments *p_T_* and *p*′*_R_* at all the expected values of *k*. The normalized cross-correlation between the two arrays is defined as:

(18) $$\rho =p_{T}⊗p_{R}^{\prime }$$

**Figure 12.**
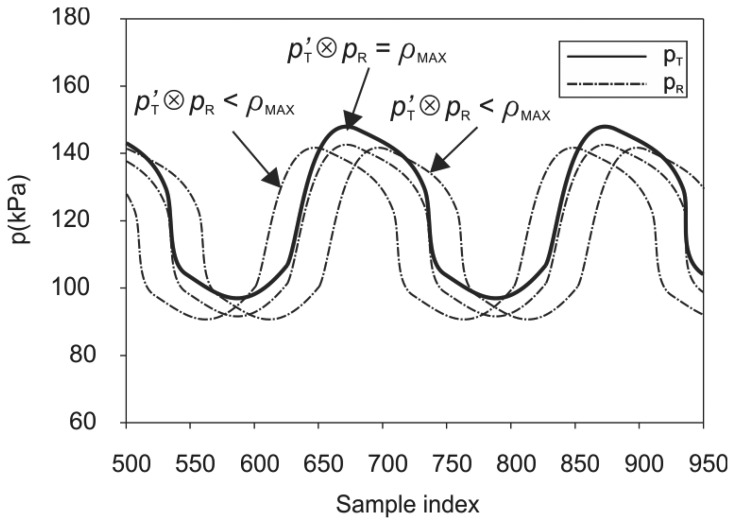
Cross-correlation between input signal and shifted output signal.

The delay *k* = *b* − *a* is defined as the lag for which the normalized cross-correlation has the largest absolute value *ρ_MAX_*. At this point, the two signals closely resemble each other. In reality, the wireless system adds some errors in amplitude. Some variation in the time delays of the individual segments is also apparent due to the dynamic clock handling of the internal buffering mechanism. The resulting small amplitude errors and time-delay deviations, however, provide sufficient correlation between the pair of the delayed signal, as shown in Figure 12.

The last step, shown in Figure 11, is the evaluation. The result of the evaluation process is a single value, calculated from the aligned signal segments for each evaluated parameter (*i.e.*, amplitude error, time delay).

The first evaluation stems from the mean difference between the signals. The difference array *δ*[*i*] is calculated by subtracting the corresponding elements from the aligned signal arrays:

(19) $$\delta \left[i\right]=\left[p_{Rc}-p_{Ta},p_{Rc+1}-p_{Ta+1},\ldots ,p_{Rd}-p_{Rb}\right]$$

The new array *δ*[*i*] is the source for the first evaluator *δ̄*. It is calculated as an average value of the array *δ*[*i*]:

(20) $$\bar{\delta }=\frac{1}{b-a}\sum_{j=a}^{b}\delta \left[j\right]$$

The signal difference evinces a systematic amplitude error. Its value represents the actual quality of the wireless transmission system. This should be kept within acceptable limits, defined by the required system accuracy.

The second evaluator is the time delay. The difference between the indices *a* and *b* is transformed into time. The time between two samples within the signal array is defined by the sampling frequency *f_S_*. The time delay *D* between the signals of the observed segments is calculated by:

(21) $$D=\frac{b-a}{f_{S}}$$

#### Acceptance Criteria for the System Evaluation

The system requirements were based on the general requirements commonly seen in industrial applications. The overall accuracy of the wireless system was set to 1% full scale. In our case, the system operated at a full scale of ±400 kPa. Accordingly, the evaluator *δ̄* should remain within ±4 kPa. In order to detect any possible anomalies a verification run of 24 hours was planned.

As regards the time delay between the signals, the requirement was to keep it below 1 s. In addition, the time delay should not change rapidly to avoid signal distortion. A separate mechanism for detection of the rapid time-delay changes is not required since the deviation would result in a significant output-signal distortion. This would consequently scale the evaluator *δ̄* above an acceptable level. Therefore, only a time delay was observed during the 24 hour test. Both acceptance criteria are shown in Table 2.

### 4.5. Test Results

#### 4.5.1. Signal Time Delay

Three sensor pairs were used during the testing, marked as sensor pairs #1, #2 and #3. The first tested parameter was the signal time delay. One transmitter was connected to three receivers at the same time and the delay between the signals was measured.

#### 4.5.2. Signal Time-Delay Variation

The internal algorithms within the communication stack compensate the time deviations between the transmitter and each of the three receivers. The transmitter/receiver pair operates at slightly different frequencies. When sampling is not exactly synchronized, one of the devices will face buffer over- or under-flow, either in acquisition or reconstruction. To overcome this problem, a dynamic time-adaptation algorithm was implemented. Since there is only one transmitter in the system, its internal clock served as the reference to all the connected receivers. Each receiver has its own buffer under- and over-flow detection algorithm. When one of the receivers runs slower than the transmitter, its reconstruction buffer gets overflowed by the samples received from the transmitter. To detect the buffer overflow, the available buffer space is evaluated. When the available buffer space is lower than a predefined level, the receiver's internal reconstruction clock is accelerated until sufficient buffer space is available. A similar situation occurs when the receiver runs faster than the transmitter. Over some time, the receiver's reconstruction buffer will underflow (*i.e.*, the transmitter will not provide enough readouts for the reconstruction). When such a situation is detected, the receiver's internal reconstruction clock is adjusted to run slower than the transmitter until the buffer recovers.

**Figure 13.**
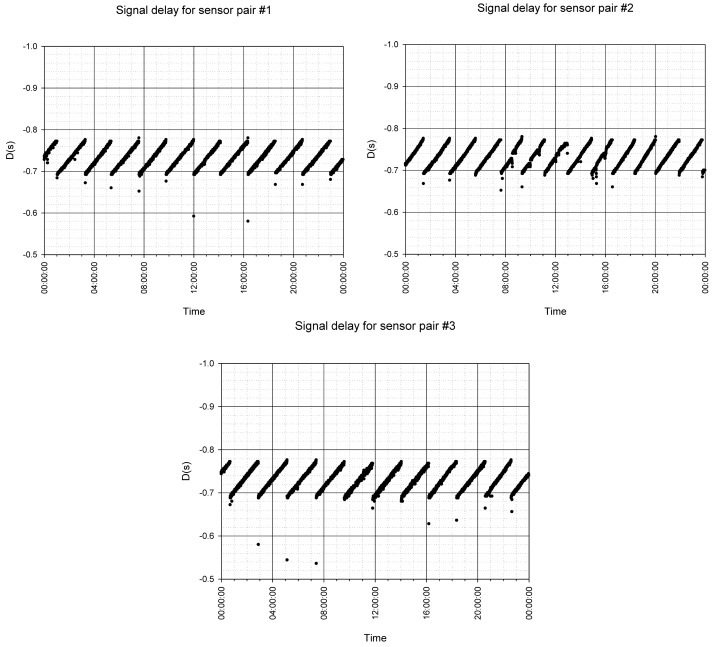
Time-delay evaluator D between p_T_ and p_R_ for 24-hour pressure measurements.

A saw-tooth shape of the time delay (Figure 13) occurs as a result of the time-deviation compensation. Notice that the measured time delay is well within the required range given in Table 2.

#### 4.5.3. System Accuracy

**Figure 14.**
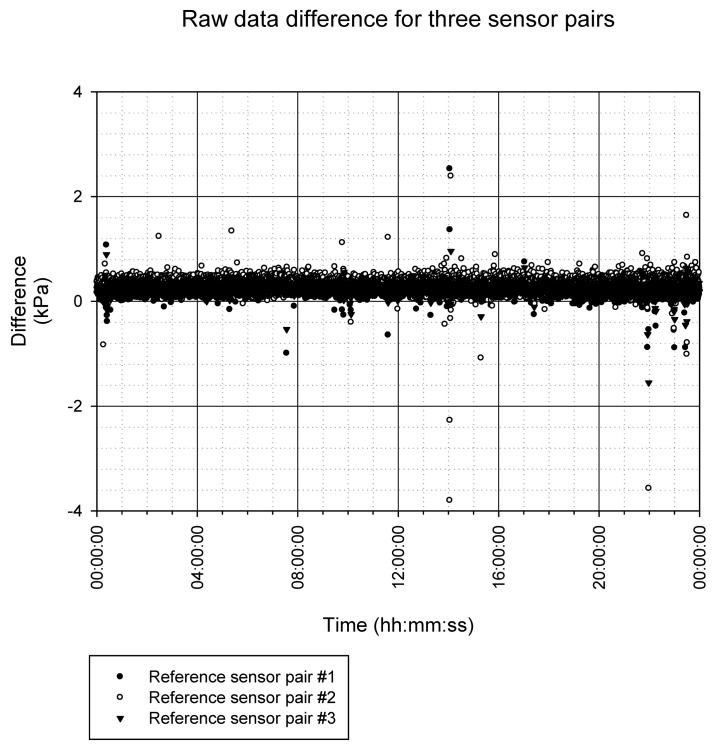
Results for evaluator δ(n) for 24-hour pressure measurements.

The average signal difference between the signals p_T_ and p_R_ (expression 20) was measured next. The results for the three sensor pairs are shown in Figure 14. The reference pressure was a periodic signal within the system's operating range. The experiment with the three receivers connected to one transmitter was running for 24 hours. Data was recorded during that time with a sampling frequency of 250 Hz. The analysis was carried out off-line. As shown in Figure 14, the requirement for the error band to be within ±4 kPa, which is ±1% of full scale, was met. The achieved statistics indicates a good system performance, even between ±1 kPa. Some sparse outliers have no impact on the target system application.

## 5. Conclusions

The main idea of this work was to investigate the possibility of implementing a wireless replacement for a sensor-bridge cable. We were able to prove the concept of an inverted topology. All the test results were obtained directly from theoretical calculations, without any additional calibration. The overall performance was within the requirements, despite a small systematic error, which was about a decade lower than the required tolerances. The concept was tested with the same results, using several receiver/transmitter pairs. This confirmed the idea of the inverted topology and validated it for mass production. The presented solution is currently integrated within a series production for a medical application.

Beyond the raw sensor-signal manipulation over the wireless link, we implemented additional mechanisms for sensor data integrity, compression and time synchronization. The data integrity mechanism keeps the transferred data valid, authentic and consistent with the process measurement. It was validated during testing. Any failure to bring consistent data via a wireless link would result in a measurable error, because the measurement system compared the received signals to the reference signals at the transmitter input. There was no error detected during 24 hours of operation, which sustained data integrity at a high level.

The data compression was based on a number-precision reduction. The internal data processing was standard double-precision floating point, while the wirelessly transmitted readouts were reduced in precision to maintain the required resolution. The advantage was a compressed data flow via a wireless link. It was tested with three simultaneous connections from a single transmitter to three receivers without any data loss.

The tolerances in the frequency of the time bases for the transmitter and the receiver resulted in asynchronous handling of the data buffers. To override any troubles with the buffer over- or under-run, we implemented a synchronization algorithm for the compensation in the time-base frequencies. The shape of the time-delay diagram follows exactly the activity of the time-synchronization algorithm. The implemented regulation model was linear. Some future investigation will be required to investigate the possibilities for implementing higher-order PID regulation or some fuzzy-logic algorithms, which could result in a smaller time-delay deviation.

The wireless technology employed was Bluetooth, which is not optimum for an agile communication administration. New and emerging wireless technologies [20,21] may provide more nimble solutions, bringing shorter delay times and lower power consumption, which will be the focus of our future work.

## Figures and Tables

**Table 1.** Evaluation of the appropriateness of the wireless technologies for various applications.

**Feature**	**CC2400** ^a^	**WT12** ^b^	**Units**
Data rate	250	723	kbps
Rx power consumption	165	231	mW
Tx power consumption	148.5	231	mW
CE, FCC qualified	Yes	Yes	
Indoor/Urban Range	30	10	m
Outdoor RF line-of-sight Range	100	(X)	m
Supported Network Topologies	Point-to-point, Point-to-multipoint, Peer-to-peer & Mesh	Point-to-point, Point-to-multipoint	
Self-routing option	Yes	(X)	
Self-healing network	Yes	(X)	
Fault-tolerant mesh network	Yes	(X)	
Supply voltage	3.3	3.3	V
UART interface	No	Yes	
SPI interface	Yes	No	
Encryption	128	128	bit
RSSI indication	Yes	Yes	
Time to connect	<0.05	>5	s
Form factor	24 × 27 × 2.3	26 × 14 × 2.3	mm
Integrated antenna	Y	Y	
External antenna	Y	N	
Price per 1k units	10.89	>20.00	€
Coexistence with other wireless technologies	Limited	Possible	

Notes:

a Source: Ti CC2400 documentation and measurements on evaluation modules;

b Source: Bluegiga WT12 documentation; X: Information not given by datasheet or user's manual.

**Table 2.** Wireless-system requirements.

**Evaluator**	**Min**	**Typ**	**Max**	**units**
Amplitude error (*δ̄*)	−4	0	4	kPa
Time delay (*D*)	0		1	s
